# Rapid growth, early maturation and short generation time in African annual fishes

**DOI:** 10.1186/2041-9139-4-24

**Published:** 2013-09-04

**Authors:** Radim Blažek, Matej Polačik, Martin Reichard

**Affiliations:** 1 Institute of Vertebrate Biology, Academy of Sciences of the Czech Republic, Květná 8 60365, Brno, Czech Republic

**Keywords:** Extreme life history, Annual fish, Explosive growth, Rapid maturation, Generation time, Killifish, Diapause, Vertebrate, Reaction norm, Savanna

## Abstract

**Background:**

Extreme environmental conditions can give rise to extreme adaptations. We document growth, sexual maturation and fecundity in two species of African annual fish inhabiting temporary savanna pools.

**Results:**

*Nothobranchius kadleci* started to reproduce at the age of 17 days and size of 31 mm and *Nothobranchius furzeri* at 18 days and 32 mm. All four study populations demonstrated rapid growth rates of up to 2.72 mm/day (23.4% of their total length). Both species may produce diapausing embryos or embryos that are able to hatch in as few as 15 days, resulting in a minimum generation time as short as only one month. Incubation on the surface of damp peat moss results in high embryo survival (73%) and a high proportion of rapidly developing embryos (58%) that skip diapauses and hatch in less than 30 days. We further demonstrated that rapid growth and maturation do not compromise subsequent fecundity.

**Conclusions:**

Our data suggest that both species have the most rapid sexual maturation and minimum generation time of any vertebrate species, and that rapid maturity does not involve paedogenesis.

## Background

Extreme environmental conditions give rise to extreme adaptations. In general, life history theory predicts that increased risk of mortality in adults selects for early maturation, high reproductive investment and short lifespan [1,2]. For example, a dry and hot climate selected for short lifespan in a Madagascan chameleon (*Furcifer labordi*) with a post-hatching lifespan of only four to five months [3], while stable, predator-free and cold conditions experienced by a small cave-dwelling aquatic salamander, olm (*Proteus anguinus*) resulted in delayed sexual maturity (16 years) with a lifespan of over 100 years and limited reproductive output [4].

**Figure 1:**
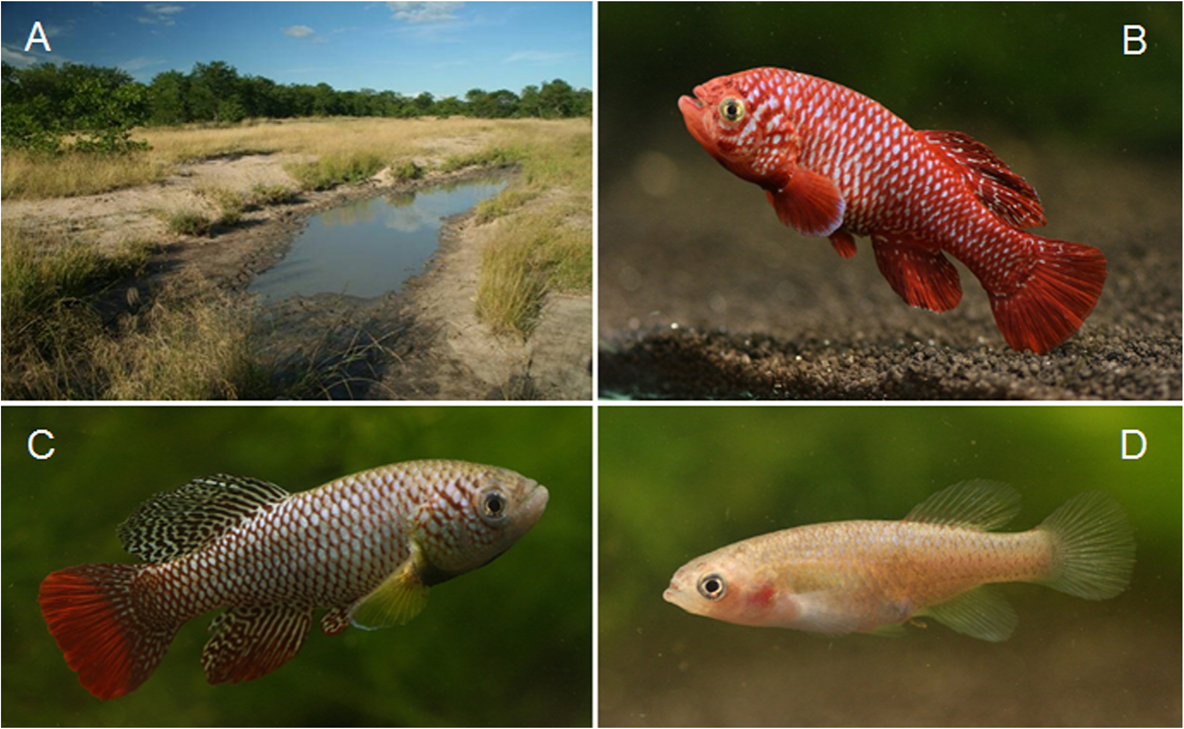
**Study species and their typical habitat. (A)** Typical habitat of the study species: a shallow savanna depression filled with water during the rainy season in Chefu region. **(B)** Male *Nothobranchius kadleci*. **(C)** Male *Nothobranchius furzeri*. **(D)** Female *Nothobranchius kadleci*.

*Nothobranchius* fishes live in extreme conditions of temporary savanna pools (Figure 1) throughout East Africa. Fish occur only during the rainy season when savanna depressions are filled with water and survive the dry season as diapaused embryos buried in soil. Annual desiccation limits their lifespan to several weeks to months [5,6] and their short lifespan is retained in captivity [7]. A population of *Nothobranchius furzeri* bred in captivity since 1969 (named GRZ) has the shortest lifespan recorded in a captive vertebrate, with a rapid increase in mortality at the age of 6 weeks, median lifespan of only 9 weeks and a maximum lifespan of 11 weeks [7].

We tested the capacity of four wild-derived populations of *N*. *furzeri* and *N*. *kadleci* for rapid growth and early maturation with their consequences for reproductive effort and embryo development under experimental conditions. *Nothobranchius furzeri* and *N*. *kadleci* are allopatric sister species inhabiting the savanna in southern Mozambique, a particularly dry part of the *Nothobranchius* range [8,9].

## Methods

Eggs of outbred F1 generation (30 wild-captured adult founders) of two populations of *N*. *furzeri* (Limpopo Basin: collection code MZCS NF121, Chefu Basin: NF222) and two populations of *N*. *kadleci* (Save Basin: NK91, Pungwe Basin: NK430) were stored at 23.5 to 24.5°C following standard culture methods [10]. Embryos were hatched in 4 L tanks with well-oxygenated tapwater (15 to 17°C). At the age of two days post-hatching, 30 individuals from each population were moved to a separate 4 L tank (temperature 24.8 to 25.8°C). At eight days fish were transferred to 60 L tanks (27 to 28°C) with internal filtration. From the age when sex was discernable (11 to 14 days) until 21 days, the sexes were separated to avoid intense male sexual harassment [11]. Fish density was reduced to four males and eight females at 21 days and two males and four females at 42 days to approximate population densities in the wild.

From 15 days to the first positive spawning record, the largest fish from each population were introduced into a 2 L tank with a spawning substrate (sand) daily (one hour, 14:00 to 15:00). After confirmation of maturation, fish were left to spawn for 2.5 hours at the age of 21, 42 and 63 days. Pilot studies demonstrated that a 2.5 h period of spawning is sufficient for oviposition of all ovulated eggs [12]. The number of eggs and fertilization success were recorded for each spawning after 24 h. As males and females were not kept separately after 21 days, females were separated from males temporarily for two days before spawning at 42 and 63 days to standardize egg production.

Fish were fed *ad libitum* with brine shrimp nauplii and weaned on to live bloodworms and *Tubifex* at age 7 days, with combined feeding at 7 to 10 days. Fish were kept under 14 h: 10 h light:dark regime in aged tap water (conductivity 550 μS.cm^-2^) throughout the experiment, with daily 30 to 50% water exchange. Fish were checked twice daily for the appearance of nuptial coloration in males. Total length (TL, from the tip of the snout to the posterior end of the caudal fin) was measured in a subsample of fish at hatching and age of 7, 14, 21, 28, 42, 63, 84 and 112 days. Fish were photographed from above in a gridded container with a thin layer of water. Measurements were made on digital photographs. Growth rate was calculated as the increase in TL and percentage of increase in TL (% TL) per individual fish per day. Body mass was not estimated. Body mass variability is large in *Nothobranchius* as they consume large amounts of food and gut fullness considerably affects body mass. The experiment was terminated at 112 days; no quantitative survival data were collected given the small number of experimental fish. The temporal change in egg production (Poisson distribution) and fertilization success (binomial distribution) were compared with a Generalized Linear Model (GLM) [12].

Data on the minimum age at sexual maturity combined with the minimum duration of embryonic development can provide information on the minimum generation time. We acknowledge that this is not an explicit estimation of generation time (for example, *sensu* Caswell 2001) [13], but it would be the actual generation time if reproduction only occurred with the earliest maturing and fastest developing individuals in laboratory conditions. The embryos of *Nothobranchius* may enter a diapause that prolongs their development or may develop without a diapause as an ‘escape’ embryo [14]. To obtain the data on the minimum duration of embryonic development in both species, the eggs of one *N*. *furzeri* population (NF121, Limpopo Basin) and two *N*. *kadleci* populations (NK91 and NK108: Save Basin) were used. The fertilized eggs were removed from spawning substrate (sand) and placed on a Petri dish filled with one of the three commonly used substrates; damp peat moss (garden peat moss Kera, http://www.kera.cz, pH 2.8 to 4.5, country of origin: Belorussia), or immersed in 5 mm deep layer of tank water or Yamamoto solution (both treated with methylene blue) [10,15]. The eggs were incubated for 30 days at 25.0 ± 0.2°C. Fully developed embryos were transferred into relatively colder tap water (16°C) when they appeared to be ready to hatch (“golden eye stage”) and their ability to hatch was confirmed [10]. The observation period was finished 30 days after the eggs were spawned. The proportion of embryos hatched in the first 30 days (that is, rapid development, skipping diapauses) and mortality rates in the first 30 days were compared across development media using survival analysis (paired log-rank tests) [16] using 25 females from three populations (NK91: 10 females, NK108: 9 females, NF121: 6 females). In mortality rate analysis, embryos surviving the study period (30 days) were censored. In developmental rate analysis, death embryos were censored at the day of their death and all embryos not hatching within 30 days period were censored at age 30 days.

The study was conducted in accordance with Czech legal requirements. Experimental procedures were approved by the ethical committees of the Institute of Vertebrate Biology, AS CR and Ministry of Agriculture, permits No. 138/2010 (project license), 065/2001-V1 (personal license), and CZ 62760203 (institutional license).

## Results

**Figure 2:**
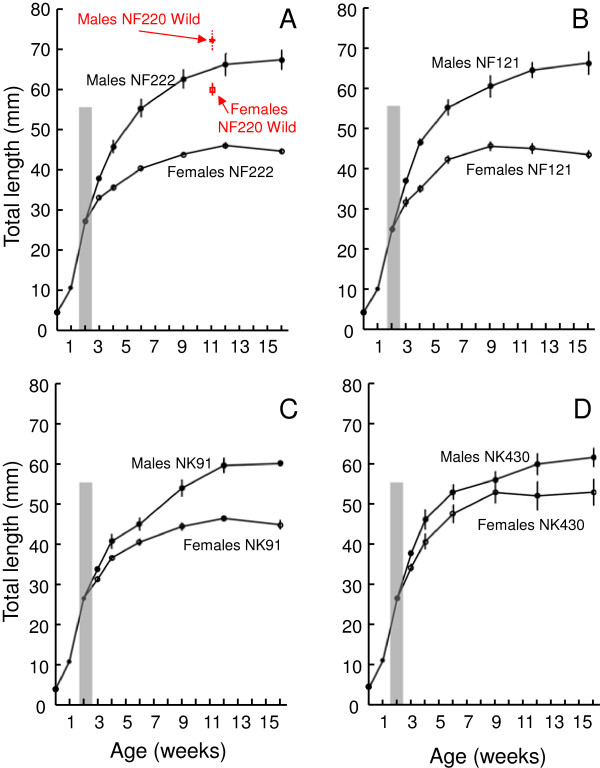
**Growth rates of all study populations.** Growth rates of the males and females for each of the four study populations of *N. furzeri* (**A**: NF222 Chefu, **B**: NF121 Limpopo) and *N. kadleci*: **C**: NK91 Save, **D**: NK430 Pungwe), with indication of the period between the onset of nuptial coloration in males and attainment of sexual maturity in both sexes (gray columns). For NF222, total length data from an adjacent population NF220 (N = 3 males, 7 females) collected in the wild are added for comparison.

All study populations demonstrated rapid growth, especially in the first 28 days. Growth decelerated from 35 days (Figure 2). Individual growth curves were constructed by measurements of the largest fish in each population. Maximum growth rates were recorded from individual growth trajectories of the largest fish between 7 and 14 days (2.72 mm (23.4% of their TL) day^-1^ in NF222, 2.34 mm (18.0% of TL) day^-1^ in NK430, 2.33 mm (19.2% of TL) day^-1^ in NK91 and 2.15 mm (17.0% of TL) day^-1^ in NF121 in the largest males).

Experimental fish attained sexual maturation at 17 to 19 days, when females measured 29 to 31 mm (Table 1, Figure 2). All early spawning events resulted in successful fertilization. Males of NF121 were repeatedly observed attempting to spawn at 16 days, but no females were mature in this population at that age. Male nuptial pigmentation appeared from 11 to 13 days and red coloration was detectable 0 to 2 days later (Table 1).

**Table 1:** **Population specific parameters of maturation, fertilization success and maximum fecundity in *Nothobranchius *fishes**

**Population identity**	**(a) Nuptial coloration**		**(b) First spawning**			**(c) 21 days Spw**	**(d) Mean ± s.e. number of eggs (N females; fertilization success in %)**				**(e) Maximum fecundity**
	**Age days**	**Length (TL, range in mm)**	**Age**	**Length (TL, mm)**							
			**Days**	**Male**	**Female**	**(in %)**	**1st Spw**	**21 days**	**42 days**	**63 days**	
NF121	13	22.9 to 24.6	19	35.2	29.0	57 (7)	12 (1; 58)	13 ± 6.5 (4; 38)	203 ± 14.2 (4; 45)	244 ± 15.8 (4; 70)	291 (63; 48.0)
NF222	12	23.6 to 25.1	18	37.9	32.2	90 (10)	2 (1; 50)	24 ± 5.5 (9; 47)	112 ± 32.9 (4; 70)	120 ± 44.6 (3; 81)	190 (63; 43.8)
NK91	12	20.9 to 24.7	17	33.6	32.4	74 (19)	6 (1; 33)	19 ± 3.8 (14; 40)	80 ± 19.4 (7; 71)	90 ± 36.9 (4; 75)	248 (84; 47.2)
NK430	11	22.1 to 22.3	17	33.6	31.3	78 (9)	17 (1; 59)	39 ± 9.9 (7; 31)	295 ± 61.3 (6; 42)	335 ± 114.8 (4; 72)	583 (63; 56.9)

Population specific parameters for age and body size at the onset of nuptial coloration (a), age of first spawning with male and female size (b), proportion of experimental females that spawned at age of three weeks (%, with sample size in parentheses) (c), mean ± s.e.m. number of eggs (with sample size and fertilization success (%) in parentheses) (d) and maximum fecundity (number of eggs, with age and female size in brackets) (e).

At the age of 21 days, 76% females (n = 45) spawned. Egg production markedly increased between 21 and 42 days, but not between 42 and 63 days (GLM with quasipoisson error structure: *F*_1,67_ = 62.7, *P* <0.001, Table 1). There were interpopulation differences (*F*_1,67_ = 20.3, *P* <0.001), with two populations (NK430 and NF121) increasing their egg production more than the other two populations (Table 1). Fertilization success gradually increased from 21 to 63 days (GLM with quasibinomial error structure: *z* = 18.6, *P* <0.001, Table 1). Maximum egg production in one 2.5-hour spawning cycle was 583 eggs for a NK430 female (63 days, 56.9 mm). In the other populations maximum egg production was 248 eggs for NK91 (84 days, 47.2 mm), 291 eggs for NF121 (63 days, 48.0 mm) and 190 eggs for NF222 (63 days, 43.8 mm).

**Figure 3:**
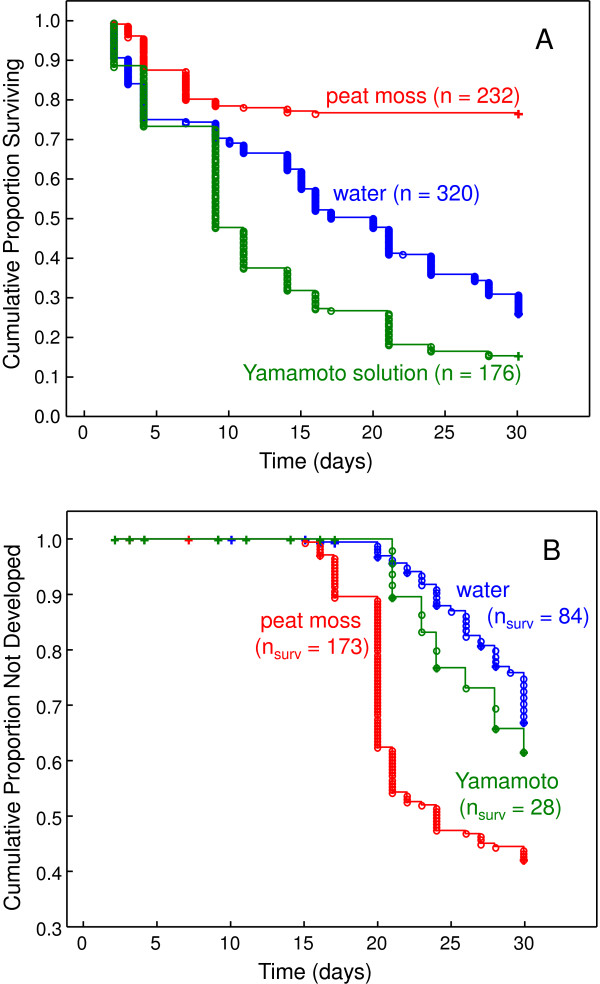
**Embryo** s**urvival and development during the first 30 days. (A)** Proportion of embryos surviving on different developmental substrates visualized by Kaplan-Meier estimate. Sample size for each category is given in parentheses. **(B)** Proportion of embryos hatched at a given time when incubated on different substrates visualized by Kaplan-Meier estimate. The number of embryos surviving the experimental period for each category is given in parentheses.

The highest survival of developing embryos was recorded on peat substrate (73% embryos survived during the first 30 days), followed by tank water (36%) and Yamamoto solution (22%) (log-rank test for survival data: *P* <0.001 for all paired comparisons) (Figure 3A). First hatching of fully developed embryos was observed after 15 days of incubation (*N*. *kadleci*, NK91) and 20 days in NK108 and NF121. The fastest embryo development was recorded on the peat substrate (paired log-rank tests: peat vs. water: *P* <0.001; peat vs Yamamoto: *P* = 0.004; water vs. Yamamoto: *P* = 0.378), with 58% of surviving embryos hatched after 30 days on peat substrate and 46% and 45% in Yamamoto solution and water, respectively (Figure 3B). The life history of hatched juveniles was not followed.

## Discussion

Rapid growth, early sexual maturation and high reproductive investment are traits typical of organisms inhabiting temporary and unpredictable habitats [1,2]. We documented extremely early maturation in two species of African annual fish. Notably, *Nothobranchius* do not compromise early sexual maturation with modification of its developmental trajectory and displayed all the morphological features typical of its evolutionary lineage [5].

Current published records of early maturity in a vertebrate are for a miniature pedomorphic goby, *Schindleria* sp. (23 days at 15 mm) [17]. Among non-pedomorphic vertebrates, female Alston’s brown mouse (*Scotinomys teguina*) achieve maturity at 28 to 39 days in the wild [18], females of some lab strains of house mouse (*Mus musculus*) mature at 23 days (although males mature much later, at the age of approximately 6 weeks) [19], a pygmy goby (*Eviota sigillata*) at 34 days [20], and the chameleon *F*. *labordi* at 2 months [3]. In other vertebrate taxa, sexual maturation typically takes considerably longer.

We demonstrated that *N*. *kadleci* can mature after 17 days at a size of 31/37 mm (female/male) and *N*. *furzeri* after 18 days at 32/38 mm (female/male). This finding is much earlier than previous reports for the GRZ laboratory strain of *N*. *furzeri* of four weeks [7]. Previous reports of early maturation in *N*. *furzeri* are based on anecdotal general statements [15]; here we provide a quantitative analysis. Further, the GRZ strain has been bred in captivity since 1969, is highly inbred [21] and likely artificially selected for traits facilitating captive breeding. In contrast, our study fish were non-inbred F2 generations of wild populations with naturally evolved life history trade-offs. Our study was designed to mimic natural conditions through *ad libitum* feeding, reducing fish density over time, and using the largest individuals to estimate rates of growth and development. In both *N*. *furzeri* and *N*. *kadleci* intense male-male competition favors high survival of the largest males and males of comparable size are frequently encountered in the wild [8,22]. The only age estimates obtained in the wild indicate that all individuals older than 27 days were sexually mature, with no younger fish included in that study [22].

All study populations of *Nothobranchius* demonstrated rapid growth, especially in the first 28 days. They also showed rapid deceleration of growth, with negligible growth after the age of 60 days (Figure 2). Maximum growth rates were recorded during their second week of life, reaching up to 2.15 to 2.72 mm (17 to 23.4% of TL) day^-1^ in the largest males. This period corresponded with a decrease in fish density, increase in water temperature (from 25 to 27.5°C), and weaning to a nutritionally richer diet, which are all features that match temporal changes in nature. After 14 days, fish growth rates decreased (Figure 2) as fish started to allocate resources to reproduction. Growth rates of *N*. *furzeri* and *N*. *kadleci* are unusually high among killifish (Cyprinodontiformes). For example, the maximum growth rate of juvenile *Austrolebias viarius* (>1 month old), a Neotropical annual killifish, achieved 0.70 mm (2.3% of TL) day^-1^ at 25°C in captivity and 0.66 mm day^-1^ in the wild (17 to 23°C) [23]. The growth rates of *N*. *furzeri* and *N*. *kadleci* are also unique within the genus [24,25]. For example, in *Nothobranchius korthausae*, a congeneric species of comparable size at hatching but considerably longer-lived (57 and 81 months for mean and maximum lifespan), fish at an age of 2 to 4 weeks are only 25% of the size of *N*. *furzeri* and *N*. *kadleci*[25]. Further, *N*. *korthausae* approach asymptotic length at the age of 40 weeks, compared to age of 10 to 12 weeks in *N*. *furzeri* and *N*. *kadleci*.

The growth rates in the wild are comparable and may be even higher. A wild population of *N. furzeri* (NF220, in close proximity to the study population NF222), with a known maximum age of 11 weeks (estimated from the fact that a logger recorded watering of the habitat 78 days prior to fish collection, see [26] for more details) attained 71.7 ± 2.1 mm (maximum 76 mm) and 60.0 ± 1.0 mm (maximum 63 mm) for males and females, respectively. The growth rates in the wild are variable and affected by fish density and prey availability [27]. However, the presented growth rates and maturation schedule are within the natural range of the studied species and favorable habitat conditions can likely promote even faster growth.

High adult mortality risk experienced by *Nothobranchius* is predicted to select for increased allocation to reproduction [1,2]. A notable outcome of the study was the high fecundity of experimental females. Egg production markedly increased between 21 and 42 days, when females still allocated a large proportion of resources to growth, but remained stable and high afterwards. Note that an increase in egg production coincided with change in experimental conditions, though we consider the effect of increasing body mass more important than a decrease in population density as food was delivered *ad libitum*. The increase in daily egg production when environmental conditions were temporarily enhanced (fresh food, partial water exchange) was reported in Neotropical annual fish, *Austrolebias nigripinnis*[28]. In general, egg production of *A. nigripinnis* is markedly lower than in *Nothobranchius*[11,12,24], with the number of eggs rarely exceeding 20 per day (typically 0 to 12 eggs) [28].

Fertilization success gradually increased from 21 to 63 days (Table 1). Maximum recorded fecundity was 583 eggs laid during 2.5 hours by *N. kadleci* female (Pungwe population) (age 63 days, 56.9 mm). *Nothobranchius* normally lay 5 to 50 eggs daily and each egg is laid singly [11]. In our setting, such fecundity was typical for females until 21 to 42 days. Once growth rate decelerated, females laid up to several hundreds of eggs, demonstrating the ability of *N. furzeri* and *N. kadleci* to elevate their energetic allocation to reproduction if conditions are favorable. Environmental conditions in *Nothobranchius* habitats vary temporally and spatially [8,26], and fecundities presented here are likely close to the upper limit of the reaction norm (that is, plastic phenotype expression of a genotype across environmental conditions) expressed under conditions of low population density and high food availability.

In this study we found a proportion of embryos to develop within 15 days at 25.0 ± 0.2°C and Valenzano *et al*. [15] provided an estimate of only 12 days for *N. furzeri* at 26°C. This results in a plausible minimum generation time of one month in both species. This interval is long compared to invertebrate models; *D. melanogaster* (10 days) and *C. elegans* (4 days), but considerably shorter than lab mice (70 to 80 days) [19]. The calculated generation time is applicable to laboratory conditions; fish follow a natural annual cycle in the wild and produce one generation each year [22]. Likewise, the other model species have much longer generation times *sensu* Caswell (2001) [13] in the wild. We also note that higher temperatures (at least 28°C) for embryo incubation are possible and should result in even shorter embryo development and, hence, generation time, though this has not been tested. Finally, we did not follow hatched fish and did not estimate the proportion of fish with insufficient swimming capacities (‘belly sliders’). They frequently occur, at least in captive conditions, including our study populations and are unable to reproduce normally. Consequently, only a study following rapidly developing fish over more generations would unequivocally demonstrate the ability of *N. kadleci* and *N. furzeri* to produce a large number of generations in rapid succession.

A further important outcome of our tests of laboratory protocols is that over 50% of embryos skipped all diapauses and developed within a short period (30 days), along with a good survival rate (73% for embryos) for embryos developing on a damp peat moss. This extraordinary short generation time and high embryo survival in the lab makes them ideal model species for aging research [29].

The natural generation time of wild *Nothobranchius* is strictly determined by filling and desiccation of their habitats and thus strongly linked to the alternation of rainy and dry seasons [5,26]. However, we believe that the ability to complete a generation within one month is adaptive. Both studied species live at a periphery of *Nothobranchius* distribution [5,30] where rainfall is unpredictable and erratic. A pool with fish may occasionally dry out and be watered again within a single rainy season. Under these circumstances the embryos with fast development may give rise to the second generation of fish that would populate a secondary pool.

## Conclusions

We demonstrated that *N. furzeri* and *N. kadleci* are species with the earliest known maturation schedule and shortest generation time among vertebrates. Their ontogeny is associated with extremely rapid growth rates and is not compromised by pedogenesis as sexually mature *Nothobranchius* fishes possess all the characteristics of typical adult teleosts. *Nothobranchius* appear to display high phenotypic plasticity with broad reaction norms for life history traits, representing an adaptation to the unpredictable conditions of seasonal pools to which they are adapted.

## Abbreviations

GLM: Generalized linear model; GRZ: Inbred captive strain of *Nothobranchius furzeri* from Gona Re Zhou Reserve; NF91: *Nothobranchius kadleci* population code MZCS91; NF108: *Nothobranchius kadleci* population code MZCS108; NF121: *Nothobranchius furzeri* population code MZCS121; NF220: *Nothobranchius furzeri* population code MZCS220; NF222: *Nothobranchius furzeri* population code MZCS222; NF430: *Nothobranchius kadleci* population code MZCS430; TL: Fish total length.

## Competing interests

The authors declare that they have no competing interests.

## Authors’ contributions

RB, MP and MR conceived and designed the study. RB and MP performed the experiments, MR performed the statistical analysis, RB, MP and MR wrote the manuscript. All authors read and approved the final manuscript.

## Authors’ information

MP and RB are postdoctoral researchers in MR’s lab working on evolutionary ecology using fish models. They are working, in particular, on the life history evolution in annual *Nothobranchius* fishes.
